# Draft genome sequence of a multidrug-resistant *Streptococcus agalactiae* sequence type 1665 strain isolated from ascitic fluid

**DOI:** 10.1128/mra.00412-26

**Published:** 2026-06-26

**Authors:** Saki Kanehira, Yusuke Ota, Kazunari Sonobe, Naoya Ichimura, Koh Okamoto, Yoshiaki Gu, Koji Sasaki, Ryoichi Saito

**Affiliations:** 1 Department of Clinical Laboratory, Institute of Science Tokyo Hospital 99038, Tokyo, Japan; 2 Department of Molecular Microbiology and Immunology, Graduate School of Medical and Dental Sciences, Institute of Science Tokyo 13290, Tokyo, Japan; 3 Department of Infectious Diseases, Graduate School of Medical and Dental Sciences, Institute of Science Tokyo 13290, Tokyo, Japan; University of Pittsburgh School of Medicine, Pittsburgh, Pennsylvania, USA

**Keywords:** multidrug-resistant *Streptococcus agalactiae*, ascitic fluid, draft genome sequence

## Abstract

*Streptococcus agalactiae* poses a threat due to increasing antimicrobial resistance and the potential for invasive disease. We isolated a sequence type 1665 strain nonsusceptible to β-lactams and resistant to macrolides, tetracyclines, and fluoroquinolones, harboring previously uncharacterized PBP2X variants, highlighting the need for genomic surveillance to guide treatment and infection control.

## ANNOUNCEMENT

*Streptococcus agalactiae* is a commensal bacterium that can cause severe infections in immunocompromised individuals, with growing concern regarding antimicrobial resistance (AMR) (1). Herein, a multidrug-resistant *S. agalactiae* TMDU1053 isolate was recovered from the ascitic fluid of a hospitalized patient at the Institute of Science Tokyo Hospital following overnight incubation at 35°C on sheep blood agar. The isolate was initially identified by MALDI Biotyper (Bruker Daltonics GmbH, Bremen, Germany). Antimicrobial susceptibility testing revealed nonsusceptibility to penicillin G and ampicillin and resistance to erythromycin, azithromycin, minocycline, and levofloxacin (2). The strain was classified as serotype Ib based on PCR analysis using previously described primers (3).

Genomic DNA was extracted from bacterial colonies grown overnight on trypticase soy agar with 5% sheep blood using a NucleoBond HMW DNA Kit (Macherey-Nagel, Düren, Germany). Library preparation was performed using the Illumina DNA Prep (M) Tagmentation Kit, and 2 × 150 bp paired-end sequencing was conducted on a NovaSeq 6000 system using the NovaSeq 6000 SP Reagent Kit v1.5 (Illumina Inc., San Diego, CA, USA). Raw short reads were quality-filtered using fastp v0.19.5 (4). Filtered reads were *de novo* assembled using Unicycler v0.5.0 (5). Genome completeness was assessed using BUSCO v6.0.0 with the bacteria_odb10 data set (6). Genome annotation was performed using Prokka v1.11 (7). Bacterial species, sequence type (ST), AMR-associated genes, and virulence genes were identified using GTDB-Tk v1.7.0 (8), the pubMLST database (9), ResFinder v4.5.0 (10), and VFDB (11), respectively. AMR-associated mutations were identified through comparison with the reference genome sequence of *S. agalactiae* 2603V/R (NC_004116). Default parameters were used unless otherwise noted.

The draft genome comprised 40 contigs totaling 2,224,547 bp with 350× coverage, an N50 of 204,312 bp, and 35.28% GC content. Genome annotation predicted 2,189 coding sequences. The genome showed 99.2% completeness, with 0.8% fragmented and no missing genes. Average nucleotide identity to the *S. agalactiae* reference strain (GCF_000186445.1) was 98.35%. The isolate was classified as ST1665 and belonged to clonal complex 1, which has been associated with invasive infections in Japan (12), although multidrug-resistant ST1665 isolates have rarely been reported. Resistome analysis revealed AMR genes conferring resistance to macrolides (*mreA*) and tetracyclines (*tetM*). Mutations associated with reduced susceptibility to penicillin were identified in PBP2X (V405A, Q557E), whereas those linked to quinolone resistance were found in GyrA (S81L) (13). Additionally, several other mutations of unknown relevance to AMR (PBP2X: G398A; GyrA: V486I) were detected. The isolate carried virulence genes for adherence (*hylB*, *lmb*, *pilC*, *scpB*, and *srtC*), hemolysis (*cyl* operon), and defense (*cps* operon), suggesting the potential to colonize host tissues and cause infection (14). Our data provide a basis for understanding AMR mechanisms and the pathogenic potential of the *S. agalactiae* ST1665 strain recovered from a clinical specimen and highlight the clinical relevance of genomic characterization for guiding treatment and infection control strategies.

## Data Availability

The accession number of the assembled genome of the *S. agalactiae* TMDU1053 strain is JBRNXA000000000. Raw sequencing data have been deposited in the Sequence Read Archive under accession number SRR35754973.
